# Examining a Common Method of Measuring Infant Fear: Considering Temperament, Neurophysiology, Age, and Sex Differences

**DOI:** 10.1002/dev.70094

**Published:** 2025-10-20

**Authors:** Joshua J. Underwood, Marco A. Ramirez Gonzalez, Kallie L. Distler, Clarissa S. Muhlestein, Maria A. Gartstein

**Affiliations:** ^1^ Washington State University Pullman Washington USA; ^2^ Baylor University Waco Texas USA

## Abstract

The pre‐locomotor version of the Laboratory Temperament Assessment Battery (Lab‐TAB) provides one of the most widely used observational measures of fear based on the infant's reactivity to a series of four novel masks. Resulting indicators of facial and bodily fear intensity, as well as latency to exhibit a fearful response, have been associated with maternal reports of infant fear as well as frontal electroencephalography (EEG) reactivity. While these measures have been used extensively since the introduction of Lab‐TAB, they are typically averaged across the procedure, and differences between the four mask stimuli have not been sufficiently examined. This study addressed this gap in research by examining specific infant reactions, both behaviorally and from a neurophysiological standpoint, to each presentation of the fear‐provoking stimuli to better understand factors that impact the expression of fear during this task. Our findings indicated significant differences in behavioral observations of distress and regulation, with the final/fourth mask eliciting stronger reactions; however, there were no such differences in the infants’ neurophysiological response. Additionally, neither the fear subscale of the Infant Behavior Questionnaire—Revised (IBQ‐R) nor the Negative Emotionality factor was predictive of infant's neurophysiological changes, distress, or regulation during the episode until examining subgroups based on age or sex. Further analyses of frontal alpha asymmetry across masks indicated that infants may become attuned to these stimuli over the course of the task, leading to less predictive utility than earlier neurophysiological markers. Results also revealed that older infants had differing neurophysiological reactions across the paradigm, whereas no significant differences were noted for younger infants. Finally, differences based on infant sex emerged with regard to temperament predictors of asymmetry. Implications of the discrepancy between results for the EEG asymmetry markers and observed distress/regulation in trial comparisons are discussed.

## 1 Introduction

Several methods have been developed to assess infant temperament and associated neurobehavioral development, with laboratory observations serving as a critical source of information. While parent report instruments are widely used to measure infants' temperament, this approach has known limitations. Parent reports of infant temperament can be biased due to factors such as social desirability, limited accuracy in the parent's memory, contrast effects, or lack of knowledge of their child's behavior (Gartstein and Rothbart 2003; Saudino 2003. Additionally, prior work by Liskola et al. (2021) revealed an association between maternal mental health and the mother's perception of their child's behavior. This “depression‐distortion” hypothesis posits that depressed mothers may show a negative bias toward their child's behavior, leading to an overestimation of distress‐related behaviors (Gartstein and Marmion 2008). To examine infant temperament in a laboratory setting, researchers often turn to emotion‐eliciting tasks, with the Laboratory Temperament Assessment Battery (Lab‐TAB; Goldsmith and Rothbart 1996a) representing the most commonly used standardized protocol. Lab‐TAB tasks were designed to elicit a broad range of emotions, including fear, anger, and positive affect/joy. In the pre‐locomotor version (recommended for infants aged 6 months; Goldsmith and Rothbart 1996a), a short exposure to a series of four novel stimuli, namely a series of masks, has become one of the most widely used episodes to examine fear reactivity (Planalp et al. 2017). Indicators derived from this task have been studied in relation to numerous factors, such as infant regulation (Wu and Gazelle 2021), cultural differences (Gartstein et al. 2016), communication (Ollas‐Skogster et al. 2023), and the gut microbiome (Carlson et al. 2021). There is some literature suggesting that the mask stimuli increase in intensity with respect to fear‐eliciting properties. That is, each mask is presented in a series, which has been described as eliciting a progressively increasing fear response (Planalp et al. 2017). However, we were not able to locate any literature addressing each of these mask presentations individually or considering potential differences in the underlying neurophysiological processes between masks.

Of note, there have been discordant findings between fear reactions elicited during this task, rated by trained experimenters/coders, and parent reports of infant temperamental fear. Specifically, parent reports of infant fear do not consistently correlate with behavioral distress observed over the course of this episode (Planalp et al. 2017; Ollas‐Skogster et al. 2023). While there have been studies that reported correlations between observed infant distress and parent reports of infant temperament, these results are scarce and generally encompass a singular area of distress expression (i.e., distress vocalizations; Gartstein et al. 2016). Finally, some work has shown negative associations between observational indicators of infant fear and corresponding parental ratings (Diaz and Bell 2012). Given the consistent use of this task to measure infant fear reactivity, the lack of concordance between infants’ behavioral reactions and maternal reports of infant fear requires further study, leveraging an additional source of information derived from neurophysiological markers.

### 1.1 Frontal Asymmetry

Electroencephalography (EEG) is a commonly used neurophysiological measure and has been regularly used to examine differences in frontal lateralization during infancy (Davidson and Fox 1989; Brooker et al. 2017; Gartstein et al. 2021). This lateralization is examined via frontal EEG power differences, wherein higher activation in the right hemisphere is indicative of more withdrawal or avoidance‐related behaviors and emotions (e.g., fear). Conversely, more activation in the left frontal hemisphere is indicative of more approach‐related motivation and emotions (e.g., joy). Based on the capability model first proposed by Coan et al. (2006), it can be expected that this hemispheric reactivity to a task mirrors the emotional demands of the situation and the emotion regulation capabilities of the individual. This can be broadly understood as a reflection of the individual's central nervous system (CNS) adaptation to the emotional demands of a task. Given that the masks episode is fear‐eliciting in nature, it would be expected that infants confronted with the novel masks would experience greater relative right‐frontal activation, which has been demonstrated in prior work (Diaz and Bell 2012; Howarth et al. 2016). Thus, there appear to be gaps in our understanding of fear reactivity in infancy at the neurophysiological level, potentially related to measurement. Some of these discrepancies may be due to the scalp sites that are utilized in infancy research, as medial frontal scalp locations (i.e., F3/F4) are the most commonly considered in frontal alpha asymmetry research (Reznick and Allen 2018); however, some work has also utilized anterior frontal (i.e., Fp1/Fp2) or lateral frontal (i.e., F7/F8) scalp sites. Notably, Diaz and Bell (2012) utilized frontal pole sites in their examination of infant fear reactivity, based on work from Buss et al. (2003) that showed significant withdrawal asymmetry in the anterior frontal scalp locations. While these sites are important to consider, working with anterior frontal EEG power brings additional challenges related to artifacts, particularly in infancy, as these sites are located directly above the eyes and are more sensitive to eyeblinks or head movement (Bell and Wolfe 2008).

### 1.2 Temperament

Temperament is considered to be relatively stable even in infancy, predicting how children respond to their environment (Rothbart and Bates 2006). As the child develops, the manifestations of temperament also evolve (Rothbart 1989). The psychobiological model proposed by Rothbart (1981) posits that temperament encompasses individual differences in motor, attentional, and emotional reactivity, and self‐regulation, driven by underlying biological mechanisms, and subject to environmental inputs and adaptations. This model has gained abundant support in infancy, with research linking both overarching and fine‐grained dimensions of temperament to underlying neurobehavioral systems. Structurally in infancy research, this model focuses on three primary overarching factors: Negative Emotionality, Surgency, and Regulation/Orienting (or Regulatory Capacity/Orienting). These factors are often considered the foundation of personality, observable in infancy and becoming more complex as the infant develops (Berdan et al. 2008). This model has been shown as robust in the context of cross‐cultural and longitudinal investigations, maintaining structural consistency across development and different cultures (Putnam et al. 2008; Cozzi et al. 2013; Evans and Rothbart 2007).

Temperament dimensions, as articulated by Rothbart's psychobiological model, have been consistently linked to later internalizing and externalizing behavioral concerns. Negative Emotionality, including infant Fear, Sadness, Distress to Limitations (or frustration), and decreased ability to lower arousal/recover from distress (falling reactivity), was shown as predictive of later internalizing and externalizing behaviors (Gartstein and Rothbart 2003, Liu et al. 2021). Temperament factors derived from the psychobiological model have also been linked to frontal alpha asymmetry, with infants higher in Negative Emotionality regularly showing greater relative right frontal activation (Smith et al. 2016). Surgency, often referred to as positive affectivity, consisting of Approach, High Intensity Pleasure, Vocal Reactivity, Activity Level, Smiling and Laughter, and Perceptual Sensitivity, was shown as a protective factor with respect to the development of internalizing behaviors, with some literature linking higher levels to an increased risk for externalizing behaviors (Berdan et al. 2008; Stifter et al. 2008; Grady et al. 2023). Similar to Negative Emotionality, Surgency has been considered in EEG asymmetry research, with infants higher in Surgency more likely to show relative left frontal dominance (Smith et al. 2016). Finally, Regulation/Orienting is broadly described as the infancy foundation for later Effortful Control closely linked with executive functions, and includes an infant's ability to be soothed, their Cuddliness, ability to attend to an object over time (i.e., duration of orienting), and enjoyment of low‐intensity/less stimulating interactions (e.g., reading a book with a parent) (Gartstein et al. 2013). Regulation/orienting is generally described as a protective factor, for example, with respect to the development of behavior problems (Gartstein et al. 2012). Higher levels of Orienting/Regulation may be a useful marker of infants’ openness to parenting interventions as it encompasses soothability (i.e., the infant's reduction of fussing/crying/etc. in response to caregivers’ use of soothing techniques; Gartstein et al. 2010; Gartstein et al. 2013).

### 1.3 Age and Sex Differences

Infants’ rapid growth over the first year of life supports significant changes in a number of functional areas. Of importance for this study, there are considerable changes in frontal alpha asymmetry in early childhood, with EEG power increasing consistently over the first few years of life, and shifts in alpha power linked to ongoing brain development (MacNeill et al. 2018; Marshall et al. 2002). Gartstein et al. (2021) reported that within‐hemisphere developmental changes in EEG became less pronounced with age, whereas development in one hemisphere influenced accelerated growth in the opposite hemisphere. Greater increases in EEG power trajectories have been reported in prior work for girls, potentially indicative of faster maturation of the supportive neural systems (Anokhin et al. 2000). For girls, stronger left hemisphere influences on the right hemisphere changes were noted, whereas for boys, right hemisphere effects emerged as more prominent (Gartstein et al. 2021). As relative left frontal activation has been associated with self‐regulation as well as approach (Goodman et al. 2013; Papousek et al. 2011), it was suggested that the tendency for left frontal activity to stimulate changes on the right could in part be responsible for the frequent finding of greater self‐regulation abilities in girls during early childhood (Else‐Quest et al. 2006; Gagne et al. 2013; Matthews et al. 2009).

Additional differences in temperament related to both sex and age have been consistently demonstrated, with some work suggesting that girls are more likely to exhibit more Negative Emotionality/behavioral inhibition during infancy, and boys demonstrating more positive affectivity and approach‐based temperament traits (e.g., activity; Gartstein and Rothbart 2003; Else‐Quest et al. 2006). Notably, these differences appear to become more significant later in infancy and as infants develop into the toddler years (Gartstein et al. 2022; Rothbart and Bates 2006).

### 1.4 Current Study

The current study sought to examine the nature of the mask task at a fine‐grained level, considering each mask individually to (1) determine if there was indeed a progression of fear reactivity across trials, considering changes in frontal EEG asymmetry as well as behavioral markers of distress and regulation; (2) ascertain potential differences among individual masks with respect to infant temperament correlates; and (3) explore potential age and sex differences in infant responses to presentations of masks and temperament correlates. Based on prior literature, it was hypothesized that infants would show increasing levels of relative right frontal activation (reflective of fear/Negative Emotionality), higher behavioral distress, and more extensive attempts to regulate when presented with each subsequent mask (H1). For the second hypothesis, we anticipated that higher levels of Negative Emotionality/Fear, both observed and ascertained via maternal report, would be predictive of greater relative right frontal activation during each mask presentation, whereas Surgency and Regulatory Capacity, as well as greater observed regulation, would predict more left frontal dominance during each mask presentation (H2). Finally, prior work has shown significant sex and age differences in temperament. We therefore predicted that frontal alpha asymmetry in response to each mask would be, in part, a function of infant age and sex, and that temperament would be predictive of asymmetry in a manner dependent on age and sex parameters (H3).

## 2 Methods

### 2.1 Participants

Mother–infant dyads (*N* = 98) were recruited from the Eastern Washington/Northern Idaho region and provided informed consent for the study as described. Children with significant medical or birth complications, including infants born preterm (< 37 weeks of gestation) and/or with identified developmental delays/disabilities, were excluded. Infants were primarily White (70%), female (58%), and with married parents (78%). Participant demographic information is included in Table 1. This study was approved by the Institutional Review Board at the corresponding author's institution.

**TABLE 1:** Sample demographic information.

Group	Sample *N*	Infant age, Mean (SD)	Maternal age, Mean (SD)	SES, Mean (SD)
Total sample	98	8.81 (1.66)	30.78 (4.78)	43.64 (24.84)
Boys	46	9.11 (1.65)	30.14 (4.61)	46.18 (25.13)
Girls	52	8.54 (1.64)	31.33 (4.92)	41.34 (24.64)
< 10 months	64	7.84 (1.08)	31.69 (4.97)	45.53 (26.14)
≥ 10 months	34	10.62 (0.85)	29.07 (3.96)	40.31 (22.42)

*Note:* Maternal socioeconomic status (SES) scores assigned corresponding to occupational prestige (Nakao and Treas 1992).

### 2.2 Measures

#### 2.2.1 Laboratory Visit

The laboratory visit began with the infant being placed into a highchair with their caregiver nearby, followed by EEG cap/electrode placement and data acquisition. Baseline EEG was recorded for 1 min while the infant watched a segment of a “Baby Einstein: Baby Mozart” video, where colorful objects are displayed as classical music plays in the background (Perone and Gartstein 2019a, 2019b). This baseline episode was intended to measure brain activity in the context of a quiet, alert state. The baseline episode was followed by the Lab‐TAB mask presentation, wherein four masks, including a witch (Mask 1), an old man (Mask 2), a vampire (Mask 3), and a gas mask (Mask 4), are displayed in this order for 10 s each, with a 5‐s recovery interval between each mask (Goldsmith and Rothbart 1996b). Mothers were instructed to remain near the infant and not react to the masks or interfere during the course of the task, unless the infant became overly distressed and required soothing.

#### 2.2.2 EEG Acquisition

Infants were seated in a highchair with an EEG cap (Cortech Solutions, Wilmington, NC, USA) placed on their head. After the cap placement, small amounts of electroconductive gel were introduced into each electrode site. A total of 32 individual “pin‐type” electrodes (BioSemi; Cortech Solutions, Wilmington, NC, USA) were then “snapped” into each corresponding site. EEG data were collected via the BioSemi Active Two amplifiers with initial screening via the BioSemi acquisition software at a sampling rate of 1024 Hz. The EEG was referenced to Cz online.

#### 2.2.3 EEG Processing

Matlab (The MathWorks, Natick, MA, USA) and subsequently the EEGLAB toolbox (Delorme and Makeig 2004) were utilized for EEG data processing, with continuous EEG down‐sampled to 256 Hz. Consistent with prior infant EEG research (Gartstein et al. 2020; Gartstein et al. 2021), a high‐pass filter at 1 Hz and a 60 Hz notch filter were applied. This filtering allows researchers to eliminate effects of any environmental electrical activity and remove slower electrical frequencies commonly seen in infant EEG (Britton et al. 2016; Cherian et al. 2009). The TrimOutlier plugin from the EEGLAB toolbox was then utilized to remove and re‐interpolate excessively noisy electrodes by examining channels that display excessive amplitude compared to the average differential specific to the current sample. Data were then re‐referenced to decrease overall amplitude across all sites while still allowing for each individual site to equally contribute to this reference point. Continuous EEG was divided into 1 s epochs with 75% overlap, with epochs rejected if the absolute voltage of any electrode exceeded 100 µV for more than 100 ms, and power values were calculated in the alpha band (6–9 Hz) for F3 and F4 electrode sites, as is routine in infant EEG work (Bell and Fox 1992; Buss et al. 2003; Degnan et al. 2011). A natural log transformation is then applied to alpha power values, with asymmetry scores calculated as the difference between the transformed power values of F4 and F3 (i.e., ln power F4 − ln power F3), as previously described (Fox 1991; Fox et al. 2001; Reznik and Allen 2018; Schmidt 2008). Lower asymmetry scores correspond with greater relative right frontal EEG activation (Hane and Fox 2006), as the alpha band is inhibitory and lower alpha values indicate greater cortical activation (Barry et al. 2009; Buss et al. 2003; Coan and Allen 2004; Klimesch et al. 2007). These procedures were completed for the initial baseline and both the total mask paradigm (i.e., Mask 1 to Mask 4 asymmetry) as well as each mask presentation individually.

### 2.3 **Infant Behavior Questionnaire—Revised** (IBQ‐R)

The IBQ‐R (Gartstein and Rothbart 2003) is a comprehensive parent‐report measure of infant temperament, consisting of 191 items that have reliably been grouped into 14 fine‐grained subscales and subsequently three overarching factors: Surgency (subscales: Approach, High Intensity Pleasure, Vocal Reactivity, Activity Level, Smiling and Laughter, and Perceptual Sensitivity), Negative Emotionality (subscales: Fear, Sadness, Distress to Limitations, and Falling Reactivity), and Orienting/Regulation (subscales: Soothability, Cuddliness, Duration of Orienting, and Low Intensity Pleasure). The IBQ‐R psychometric properties are generally good, with Cronbach's *α* values ranging from 0.77 to 0.96 (Gartstein et al. 2005; Parade and Leerkes 2008). In the current sample, *α* ranged primarily from 0.78 (Duration of Orienting) to 0.90 (Fear). However, two scales fell below 0.70, with Activity Level's *α* = 0.69 and Soothability's *α* = 0.57. Evidence for the predictive and construct validity of IBQ‐R scores has been demonstrated consistently in prior research (Gartstein and Marmion 2008).

### 2.4 Distress and Regulation Coding

Infant distress and regulation behaviors during each mask were subsequently coded for indicators of infant distress, specifically vocal distress, which is coded on a 1–5 scale, ranging from neutral vocalizations to continuous crying/screaming. Distressed facial expressions were coded based on the FACS scale (Ekman and Oster 1979) on a 1–3 range dependent on the number of areas of the face that appear to manifest distress, with higher scores reflecting more significant distress. Self‐regulatory behaviors were also coded based on frequency during this period (e.g., thumb sucking, reaching for mom, table banging, self‐touching). Infants’ attempts to reach a stimulus item (e.g., the infant reaches out for the mask) were also coded on a frequency basis. Inter‐rater reliability was good, with coders achieving Kappa values of 0.80 or higher on a portion of cross‐coded videos. Composites of distress intensity were formed based on total facial and vocal distress levels across each segment of the task (*r*’s 0.40–0.58; *p* < 0.001). Regulation composites were created based on the various regulatory behaviors exhibited as a count of the total number of coded instances, with higher values representing more extensive attempts to regulate.

### 2.5 Analyses

To examine Hypothesis 1 (H1), three one‐way repeated measures ANOVAs were performed, examining differences in observed distress, regulation, and frontal alpha asymmetry across all four mask presentations. Bonferroni corrections were planned for significant ANOVAs to further examine significant differences within the mask paradigm. To further evaluate changes in frontal alpha asymmetry across masks, hierarchical regressions were subsequently conducted. The initial regression examined infant age in months, sex, and baseline asymmetry as predictors for the first mask presentation. These variables were included as a block in subsequent equations, wherein each prior mask was added as a predictor (e.g., age, sex, baseline asymmetry, and Mask 1 asymmetry predicting Mask 2 asymmetry). Age and sex were statistically controlled in these models, given their notable impacts on EEG asymmetry and their anticipated role in contributing to individual differences in response to this task. As noted, there are variations in frontal scalp sites used in infant EEG research; thus, asymmetry scores based on anterior and lateral frontal sites were examined via a similar repeated measures ANOVA for a more comprehensive evaluation of differences across mask presentations. If significant differences were observed for additional electrode locations, these would be considered further.

To examine the relationship between temperament and infant EEG asymmetry (H2), hierarchical linear regressions controlling for age, sex, and baseline asymmetry were performed for the three temperament factors, with subsequent post hoc examinations of relevant subscales for factors making a significant contribution, to further examine attributes driving asymmetry changes across the masks paradigm. Finally, to examine Hypothesis 3 (H3), the total sample was split into two groups based on the age median split, then recombined and split into two groups based on sex only. Moderation analyses of age × sex were not conducted, given the small cell sizes when the sample was split by age and sex simultaneously. Four one‐way repeated ANOVAs were performed to examine significant differences in infant asymmetry based on age and subsequently based on sex, with Bonferroni corrections following significant results. Additional regression analyses were conducted to examine the contribution of temperament predictors to EEG asymmetry for each of these groups (i.e., older and younger infants; boys and girls), with subsequent subscale analyses of significant factors.

## 3 Results

With regard to Hypothesis 1 (H1; Table 2), there were no significant differences in infant asymmetry observed across any of the four mask presentations based on the ANOVA results, indicating no significant differences in the neurophysiology of fear reactivity across individual masks. Follow‐up ANOVA results examining differences across masks at differing frontal alpha asymmetry sites also indicated no significant differences in scalp sites across each mask. Given the lack of significant differences across the frontal sites in these analyses, F3 and F4 were the only locations used to calculate asymmetry in testing the remaining hypotheses (H2 and H3), as these are primarily used in frontal alpha asymmetry research (Coan and Allen 2004; Fox 1991). Additionally, for Hypothesis 1, results from the ANOVA for observed distress were significant (*p* < 0.001, *ηp* ^2^ = 0.19). Post hoc comparisons with Bonferroni corrections (Table 3) revealed that infant distress during Mask 4 (gas mask) was significantly greater than during any other mask presentation (*p* < 0.001); however, no other trials were significantly different from each other. With regard to the ANOVA examining regulation, results were also significant (*p* = 0.01, *ηp* ^2^ = 0.08), with Bonferroni‐adjusted follow‐up comparisons revealing that during Mask 4, infants were engaged in more active regulation than during either Mask 2 or Mask 3 (*p* < 0.05) but not Mask 1. No other mask presentations were significantly different with respect to observed regulation. To further examine Hypothesis 1, hierarchical regressions were performed (Table 4) and showed that baseline asymmetry was significant in predicting asymmetry during the first mask presentation; however, it was not significant in predicting any other asymmetry scores including total asymmetry scores. Asymmetry at Mask 1 was predictive of asymmetry at Mask 2 and Mask 3; however, only asymmetry at Mask 3 was predictive of asymmetry at Mask 4. Thus, while evidence for significant differences in the frontal asymmetry response across masks was not obtained, these emerged for both infant distress and regulation, pointing toward differential reactions at the end of masks administration. In addition, temporal proximity largely determined the predictive utility of prior frontal alpha asymmetry scores. Notably, infant age was also a significant predictor of asymmetry during the fourth mask presentation, with older infants showing more right frontal activity.

**TABLE 2:** One‐way repeated measures ANOVA examining differences in asymmetry, distress, regulation, and frontal scalp sites (H1).

Variable	df	Mean square	*F*	Sig	Partial eta
Asymmetry	3	0.08	0.91	0.44	0.02
Distress Severity*	3	287.37	17.75	< 0.001	0.19
Regulation Composite*	3	41.91	7.37	< 0.01	0.09
Frontal scalp asymmetry Mask 1	2	0.06	0.40	0.67	0.01
Frontal scalp asymmetry Mask 2	2	0.17	1.04	0.36	0.02
Frontal scalp asymmetry Mask 3	2	0.24	2.04	0.13	0.03
Frontal scalp asymmetry Mask 4	2	0.26	1.77	0.18	0.03

*Indicates that Mauchley's test of sphericity was significant and thus Greenhouse–Geisser effects are reported.

**TABLE 3:** Bonferroni correction results for Hypothesis 1 significant ANOVA results (H1).

Variable	Pair	Mean difference	Std. error	Sig
Distress	Mask 1–Mask 2	−0.38	0.34	1.00
	Mask 1–Mask 3	−0.06	0.30	1.00
	Mask 1–Mask 4	−3.04	0.63	< 0.001
	Mask 2–Mask 3	0.31	0.28	1.00
	Mask 2–Mask 4	−2.66	0.60	< 0.001
	Mask 3–Mask 4	−2.97	0.63	< 0.001
Regulation	Mask 1–Mask 2	0.12	0.10	1.00
	Mask 1–Mask 3	0.05	0.09	1.00
	Mask 1–Mask 4	−0.87	0.34	0.07
	Mask 2–Mask 3	−0.06	0.09	1.00
	Mask 2–Mask 4	−0.99	0.33	0.02
	Mask 3–Mask 4	−0.92	0.33	0.04

**TABLE 4:** Hierarchical regressions examining change in asymmetry (H1).

Model step	Model statistics: Predicting Mask 1 asymmetry				
Step 1		*R* ^2^	*F*	*p*	
		0.25	6.22	< 0.01	
Predictors	Predictor statistics				
	*β*	*b*	SE*_b_*	*t*	*p*
**Baseline Asym**	**0.33**	**0.77**	**0.29**	**2.64**	**0.01**
Child Age	−0.10	−0.03	0.04	−0.49	0.43
Child Sex	0.25	0.24	0.12	1.92	0.06
	Model statistics: Predicting Mask 2 asymmetry				
Step 2		Δ*R* ^2^	Δ*F*	*p*	
		0.07	3.92	0.11	
Predictors	Predictor statistics				
	*β*	*b*	SE*_b_*	*t*	*p*
Baseline Asym	−0.04	−0.05	0.20	−0.23	0.82
Child Age	−0.17	−0.03	0.02	−1.17	0.25
Child Sex	0.03	0.01	0.09	0.16	0.87
**Mask 1 Asym**	**0.31**	**0.19**	**0.10**	**1.98**	**0.05**
	Model statistics: Predicting Mask 3 asymmetry				
Step 3		Δ*R* ^2^	Δ*F*	*p*	
		0.01	0.68	0.03	
Predictors	Predictor statistics				
	*β*	*b*	SE*_b_*	*t*	*p*
Baseline Asym	0.08	0.12	0.23	0.51	0.61
Child Age	0.14	0.03	0.03	0.93	0.36
Child Sex	0.06	0.04	0.10	0.40	0.70
**Mask 1 Asym**	**0.42**	**0.29**	**0.11**	**2.58**	**0.01**
Mask 2 Asym	0.12	0.14	0.17	0.82	0.42
	Model statistics: Predicting Mask 4 asymmetry				
Step 4		Δ*R* ^2^	Δ*F*	*p*	
		0.14	8.16	< 0.01	
Predictors	Predictor statistics				
	*β*	*b*	SE*_b_*	*t*	*p*
Baseline Asym	0.12	0.02	0.26	0.08	0.94
**Child Age**	**−0.35**	**−0.08**	**0.03**	**−2.34**	**0.03**
Child Sex	0.04	0.03	0.11	0.24	0.81
Mask 1 Asym	0.08	0.06	0.13	0.47	0.64
Mask 2 Asym	0.01	0.01	0.18	0.04	0.97
**Mask 3 Asym**	**0.42**	**0.47**	**0.16**	**2.86**	**< 0.01**

In examination of Hypothesis 2 (H2), results from hierarchical linear regressions examining temperament predictors (Tables 5 and 6) revealed that Surgency was a significant predictor of left frontal asymmetry during the first mask (*β* = 0.30, *p* < 0.05), with follow‐up subscale analyses showing no significant/unique predictors. Negative emotionality was a significant predictor of left frontal asymmetry for Mask 2 (*β* = 0.28, *p* < 0.05), with subscale analysis revealing Fear as the primary significantly predictive subscale (*β* = 0.30, *p* < 0.05), which was not anticipated. For the third mask, no significant temperament predictors emerged. Finally, for Mask 4, and again contrary to our hypothesis, Surgency was predictive of relative right frontal activation (*β* = −0.41, *p* = 0.01), with subscale analysis showing no significant predictors.

**TABLE 5:** Hierarchical regressions examining temperament factor predictors of asymmetry (H2).

Model step	Model statistics: Predicting Mask 1 asymmetry				
Step 2		Δ*R* ^2^	Δ*F*	*p*	
		0.11	2.70	< 0.01	
Predictors	Predictor statistics				
	*β*	*b*	SE*_b_*	*t*	*p*
**Baseline Asym**	**0.47**	**1.26**	**0.36**	**3.48**	**< 0.01**
Child Age	−0.17	−0.05	0.04	−1.28	0.21
Child Sex	0.13	0.13	0.13	0.99	0.33
**SUR**	**0.30**	**0.05**	**0.02**	**2.03**	**< 0.05**
NEG	−0.09	−0.02	0.03	−0.70	0.49
RCO	0.12	0.03	0.04	0.81	0.42
	Model statistics: Predicting Mask 2 asymmetry				
Step 2		Δ*R* ^2^	Δ*F*	*p*	
		0.08	2.11	< 0.001	
Predictors	Predictor statistics				
	*β*	*b*	SE*_b_*	*t*	*p*
**Baseline Asym**	**0.51**	**0.72**	**0.18**	**3.98**	**< 0.001**
Child Age	−0.14	−0.03	0.03	−1.05	0.30
Child Sex	−0.02	−0.02	0.09	−0.17	0.87
SUR	−0.06	−0.01	0.02	−0.43	0.67
**NEG**	**0.28**	**0.04**	**0.02**	**2.05**	**< 0.05**
RCO	0.25	0.04	0.02	1.84	0.07
	Model statistics: Predicting Mask 3 asymmetry				
Step 2		Δ*R* ^2^	Δ*F*	*p*	
		0.05	1.35	< 0.01	
Predictors	Predictor statistics				
	*β*	*b*	SE*_b_*	*t*	*p*
**Baseline Asym**	**0.50**	**0.80**	**0.20**	**4.12**	**< 0.001**
Child Age	0.08	0.02	0.04	0.58	0.57
Child Sex	0.16	0.13	0.10	1.28	0.21
SUR	−0.03	0.00	0.02	−0.22	0.83
NEG	0.26	0.04	0.02	1.89	0.07
RCO	0.16	0.04	0.03	1.13	0.26
	Model statistics: Predicting Mask 4 asymmetry				
Step 2		Δ*R* ^2^	Δ*F*	*p*	
		0.13	2.71	< 0.01	
Predictors	Predictor statistics				
	*β*	*b*	SE*_b_*	*t*	*p*
**Baseline Asym**	**0.40**	**0.63**	**0.21**	**3.07**	**< 0.01**
Child Age	0.17	0.04	0.04	1.13	0.26
Child Sex	0.06	0.05	0.11	0.45	0.65
**SUR**	**−0.41**	**−0.05**	**0.02**	**−2.69**	**< 0.05**
NEG	−0.06	−0.01	0.02	−0.42	0.68
RCO	0.26	0.05	0.03	1.70	0.10

Abbreviations: SUR—Surgency, NEG—Negative Emotionality, RCO—Regulation/Orienting.

**TABLE 6:** Hierarchical regressions examining temperament subscale predictors of asymmetry (H2).

Model step	Model statistics: Predicting Mask 1 asymmetry				
Step 2		Δ*R* ^2^	Δ*F*	*p*	
		0.13	1.53	< 0.01	
Predictors	Predictor statistics				
	*β*	*b*	SE*_b_*	*t*	*p*
**Baseline Asym**	**0.45**	**1.21**	**0.37**	**3.24**	**< 0.001**
Child Age	−0.22	−0.06	0.04	−1.54	0.13
Child Sex	0.18	0.18	0.13	1.38	0.18
Activity	0.10	0.07	0.10	0.76	0.45
Smiling/Laughter	−0.01	−0.01	0.09	−0.07	0.95
High Intensity Pleasure	0.31	0.25	0.15	1.70	0.10
Perceptual Sensitivity	0.06	0.03	0.08	0.39	0.70
Approach	−0.10	−0.07	0.11	−0.63	0.53
Vocal Reactivity	0.12	0.07	0.11	0.64	0.53
	Model statistics: Predicting Mask 2 asymmetry				
Step 2		Δ*R* ^2^	Δ*F*	*p*	
		0.09	1.63	< 0.001	
Predictors	Predictor statistics				
	*β*	*b*	SE*_b_*	*t*	*p*
**Baseline Asym**	**0.51**	**0.71**	**0.17**	**4.24**	**< 0.001**
Child Age	−0.24	−0.05	0.03	−1.61	0.11
Child Sex	−0.05	−0.03	0.10	−0.31	0.76
Distress to Limitations	0.04	0.02	0.06	0.25	0.80
**Fear**	**0.31**	**0.13**	**0.05**	**2.40**	**< 0.05**
Falling Reactivity	−0.06	−0.02	0.06	−0.41	0.68
Sadness					
	Model statistics: Predicting Mask 1 asymmetry				
Step 2		Δ*R* ^2^	Δ*F*	*p*	
		0.14	1.46	< 0.05	
Predictors	Predictor statistics				
	*β*	*b*	SE*_b_*	*t*	*p*
**Baseline Asym**	**0.39**	**0.62**	**0.21**	**2.89**	**< 0.05**
Child Age	0.10	0.03	0.04	0.59	0.56
Child Sex	0.20	0.16	0.11	1.41	0.17
Activity	−0.25	−0.14	0.09	−1.62	0.11
Smiling/Laughter	−0.01	0.00	0.08	−0.06	0.96
High Intensity Pleasure	−0.13	−0.09	0.14	−0.69	0.50
Perceptual Sensitivity	0.25	0.11	0.08	1.29	0.20
Approach	−0.09	−0.06	0.11	−0.50	0.62
Vocal Reactivity	−0.22	−0.11	0.10	−1.08	0.29

In the examination of Hypothesis 3 (H3), ANOVA results (Tables 7 and 8) indicated that infants 10–14 months old had significantly different asymmetry values associated with mask presentations compared to younger infants (*n* = 34; *p* < 0.05, *ηp* ^2^ = 0.16). Post hoc tests with Bonferroni corrections showed that there were significant differences associated with their response to Mask 3 and Mask 4 (*p <* 0.05); specifically, there were higher levels of right frontal asymmetry during the final mask than during Mask 3. No other infant age or sex group‐related effect emerged. Hierarchical multiple linear regressions were conducted to examine temperament predictors of infant asymmetry in younger and older age groups (Tables 9 and 10), with Surgency significantly predicting left frontal asymmetry during Mask 1 (*β* = 0.58, *p* < 0.05), and Negative Emotionality significantly predictive of right frontal asymmetry during Mask 4 for infants 10 months and older (*β* = −0.69, *p* < 0.05). Subsequent scale‐level analyses showed no significant/unique predictors of Mask 1 asymmetry, with Fear and Sadness emerging as the primary predictors of right frontal asymmetry during the fourth mask presentation (Fear: *β* = −0.81, *p* < 0.01; Sadness: *β* = −0.65, *p* < 0.01). For infants under 10 months of age (*n* = 63), Surgency was predictive of right frontal asymmetry during the fourth mask presentation (*β* = −0.49, *p* < 0.01), and Negative Emotionality was predictive of left frontal asymmetry during the third mask presentation (*β* = 0.38, *p* < 0.05). No significant results were observed at the subscale level. As for infant sex (Tables 11 and 12), no significant differences emerged across asymmetry scores for each mask presentation. For girls, Regulation/Orienting scores were significant predictors of left frontal asymmetry during Mask 2 (*β* = 0.53, *p* < 0.01), and Surgency was predictive of right frontal asymmetry during Mask 4 (*β* = −0.82, *p* < 0.001). For subscale analysis, Cuddliness emerged as a significant predictor of left frontal asymmetry during Mask 2 (*β* = 0.57, *p* < 0.01), with no significant Surgency scale predictors noted for Mask 4. No significant temperament predictors were observed for boys’ asymmetry scores.

**TABLE 7:** One‐way repeated measures ANOVA examining differences in asymmetry by age and sex (H3).

Variable	df	Mean square	*F*	Sig	Partial eta
Asymmetry: 6–9 months	3	0.20	0.23	0.88	0.01
Asymmetry: 10–14 months*	3	0.32	3.54	< 0.05	0.13
Asymmetry: Boys	3	0.21	1.69	0.17	0.07
Asymmetry: Girls	3	0.01	0.09	0.97	0.00

*Indicates that Mauchley's test of sphericity was significant and thus Greenhouse–Geisser effects are reported.

**TABLE 8:** Bonferroni correction results for Hypothesis 1 significant ANOVA results (H1).

Variable	Pair	Mean difference	Std. error	Sig
10–14 month Asym	Mask 1–Mask 2	−0.21	0.11	0.40
	Mask 1–Mask 3	−0.23	0.08	0.06
	Mask 1–Mask 4	−0.04	0.10	1.00
	Mask 2–Mask 3	−0.03	0.28	1.00
	Mask 2–Mask 4	0.16	0.09	1.00
	Mask 3–Mask 4	0.19	0.06	< 0.05

**TABLE 9:** Hierarchical regressions examining temperament factor predictors of asymmetry by infant age group (H3).

Model step	Model statistics: Predicting Mask 1 asymmetry (6–9 months)				
Step 2		Δ*R* ^2^	Δ*F*	*p*	
		0.06	0.90	0.06	
Predictors	Predictor statistics				
	*β*	*b*	SE*_b_*	*t*	*p*
**Baseline Asym**	**0.38**	**1.02**	**0.45**	**2.27**	**< 0.05**
Child Sex	0.19	0.19	0.16	1.14	0.26
SUR	0.06	0.01	0.03	0.33	0.74
NEG	−0.04	−0.01	0.04	−0.21	0.84
RCO	0.21	0.06	0.05	1.14	0.26
	Model statistics: Predicting Mask 1 asymmetry (10–14 months)				
Step 2		Δ*R* ^2^	Δ*F*	*p*	
		0.27	2.23	0.08	
Predictors	Predictor statistics				
	*β*	*b*	SE*_b_*	*t*	*p*
**Baseline Asym**	**0.75**	**2.01**	**0.86**	**2.35**	**< 0.05**
Child Sex	−0.10	−0.10	0.32	−0.32	0.76
**SUR**	**0.59**	**0.09**	**0.04**	**2.25**	**< 0.05**
NEG	−0.06	−0.01	0.06	−0.24	0.81
RCO	0.17	0.04	0.07	0.60	0.56
	Model statistics: Predicting Mask 2 asymmetry (6–9 months)				
Step 2		Δ*R* ^2^	Δ*F*	*p*	
		0.07	1.32	< 0.01	
Predictors	Predictor statistics				
	*β*	*b*	SE*_b_*	*t*	*p*
**Baseline Asym**	**0.57**	**0.84**	**0.24**	**3.51**	**< 0.001**
Child Sex	0.09	0.07	0.15	0.50	0.62
SUR	−0.06	−0.01	0.02	−0.39	0.70
NEG	0.28	0.04	0.03	1.35	0.19
RCO	0.30	0.07	0.04	1.84	0.08
	Model statistics: Predicting Mask 2 asymmetry (10–14 months)				
Step 2		Δ*R* ^2^	Δ*F*	*p*	
		0.27	1.62	0.42	
Predictors	Predictor statistics				
	*β*	*b*	SE*_b_*	*t*	*p*
Baseline Asym	−0.45	−0.51	0.43	−1.18	0.26
**Child Sex**	0.44	0.18	0.14	1.24	0.24
SUR	−0.69	−0.04	0.02	−2.15	0.05
NEG	−0.03	0.00	0.03	−0.11	0.91
RCO	−0.09	−0.01	0.03	−0.26	0.80
	Model statistics: Predicting Mask 3 asymmetry (6–9 months)				
Step 2		Δ*R* ^2^	Δ*F*	*p*	
		0.12	2.88	< 0.001	
Predictors	Predictor statistics				
	*β*	*b*	SE*_b_*	*t*	*p*
**Baseline Asym**	**0.54**	**0.85**	**0.19**	**4.47**	**< 0.001**
Child Sex	0.17	0.15	0.11	1.28	0.21
SUR	−0.18	−0.02	0.02	−1.33	0.19
**NEG**	**0.38**	**0.07**	**0.03**	**2.56**	**< 0.05**
RCO	0.14	0.03	0.03	0.97	0.34
	Model statistics: Predicting Mask 3 asymmetry (10–14 months)				
Step 2		Δ*R* ^2^	Δ*F*	*p*	
		0.27	1.62	0.29	
Predictors	Predictor statistics				
	*β*	*b*	SE*_b_*	*t*	*p*
Baseline Asym	0.18	0.36	0.76	0.48	0.64
Child Sex	0.19	0.14	0.26	0.55	0.59
SUR	0.12	0.01	0.04	0.31	0.77
NEG	−0.35	−0.07	0.06	−1.28	0.23
RCO	0.34	0.06	0.07	0.92	0.37
	Model statistics: Predicting Mask 4 asymmetry (6–9 months)				
Step 2		Δ*R* ^2^	Δ*F*	*p*	
		0.20	3.18	< 0.01	
Predictors	Predictor statistics				
	*β*	*b*	SE*_b_*	*t*	*p*
**Baseline Asym**	**0.42**	**0.51**	**0.18**	**2.78**	**< 0.05**
Child Sex	−0.05	−0.04	0.12	−0.30	0.77
**SUR**	**−0.49**	**−0.05**	**0.02**	**−2.92**	**< 0.05**
NEG	0.17	0.02	0.02	1.01	0.32
RCO	0.27	0.05	0.03	1.57	0.13
	Model statistics: Predicting Mask 4 asymmetry (10–14 months)				
Step 2		Δ*R* ^2^	Δ*F*	*p*	
		0.43	3.67	0.06	
Predictors	Predictor statistics				
	*β*	*b*	SE*_b_*	*t*	*p*
Baseline Asym	−0.17	−0.10	0.29	−0.69	0.51
Child Sex	0.35	−0.54	0.79	1.34	0.21
SUR	−0.17	0.33	0.25	−0.77	0.46
**NEG**	**−0.69**	**−0.02**	**0.03**	**−2.88**	**< 0.05**
RCO	0.14	−0.18	0.06	0.53	0.61

Abbreviations: SUR—Surgency, NEG—Negative Emotionality, RCO—Regulation/Orienting.

**TABLE 10:** Hierarchical regressions examining temperament subscale predictors of asymmetry by infant age group (H3).

Model step	Model statistics: Predicting Mask 1 asymmetry (10–14 months)				
		Δ*R* ^2^	Δ*F*	*p*	
		0.43	1.98	0.11	
Predictors	Predictor statistics				
	*β*	*b*	SE*_b_*	*t*	*p*
**Baseline Asym**	0.75	0.10	0.41	2.10	0.07
Child Sex	0.10	0.29	0.19	0.25	0.81
Activity	0.43	−0.08	0.36	1.55	0.16
Smiling/Laughter	−0.12	0.54	0.37	−0.21	0.84
High Intensity Pleasure	0.59	0.07	0.17	1.44	0.18
Perceptual Sensitivity	0.15	−0.12	0.25	0.44	0.67
Approach	−0.16	−0.02	0.22	−0.47	0.65
Vocal Reactivity	−0.03	0.10	0.41	−0.10	0.92
Model step	Model statistics: Predicting Mask 3 asymmetry (6–9 months)				
		Δ*R* ^2^	Δ*F*	*p*	
		0.10	1.73	< 0.001	
Predictors	Predictor statistics				
	*β*	*b*	SE*_b_*	*t*	*p*
**Baseline Asym**	**0.57**	**0.18**	**0.12**	**4.55**	**< 0.001**
Child Sex	0.21	0.00	0.09	1.46	0.15
Distress to Limitations	0.00	0.06	0.07	0.01	0.99
Fear	0.10	−0.08	0.07	0.78	0.44
Falling Reactivity	−0.18	0.10	0.08	−1.22	0.23
Sadness	0.20	0.18	0.12	1.21	0.24
Model step	Model statistics: Predicting Mask 4 asymmetry (6–9 months)				
		Δ*R* ^2^	Δ*F*	*p*	
		0.21	1.52	< 0.05	
Predictors	Predictor statistics				
	*β*	*b*	SE*_b_*	*t*	*p*
**Baseline Asym**	**0.43**	**0.53**	**0.19**	**2.72**	**< 0.05**
Child Sex	0.01	0.01	0.13	0.06	0.95
Activity	−0.17	−0.09	0.10	−0.96	0.34
Smiling/Laughter	−0.17	−0.07	0.09	−0.76	0.46
High Intensity Pleasure	−0.25	−0.14	0.15	−0.98	0.34
Perceptual Sensitivity	0.23	0.09	0.09	1.04	0.31
Approach	−0.25	−0.14	0.11	−1.25	0.23
Vocal Reactivity	0.02	0.01	0.12	0.08	0.93
Model step	Model statistics: Predicting Mask 4 asymmetry (10–14 months)				
		Δ*R* ^2^	Δ*F*	*p*	
		0.65	8.02	< 0.01	
Predictors	Predictor statistics				
	*β*	*b*	SE*_b_*	*t*	*p*
Baseline Asym	−0.45	−1.46	0.67	−2.18	0.05
**Child Sex**	**0.77**	**0.72**	**0.18**	**4.10**	**< 0.001**
Distress to Limitations	−0.45	−0.38	0.23	−1.62	0.14
**Fear**	**−0.81**	**−0.42**	**0.12**	**−3.60**	**< 0.001**
Falling Reactivity	−0.18	−0.11	0.15	−0.71	0.49
**Sadness**	**−0.65**	**−0.35**	**0.09**	**−3.79**	**< 0.001**

**TABLE 11:** Hierarchical regressions examining temperament factor predictors of asymmetry by infant sex (H3).

Model step	Model statistics: Predicting Mask 1 asymmetry (boys)				
		Δ*R* ^2^	Δ*F*	*p*	
		0.20	1.78	0.15	
Predictors	Predictor statistics				
	*β*	*b*	SE*_b_*	*t*	*p*
Baseline Asym	0.26	1.49	1.19	1.26	0.23
Child Age	−0.18	−0.08	0.10	−0.76	0.46
SUR	0.44	0.14	0.07	1.94	0.07
NEG	−0.16	−0.04	0.06	−0.72	0.48
RCO	0.01	0.00	0.09	0.04	0.97
	Model statistics: Predicting Mask 1 asymmetry (girls)				
		Δ*R* ^2^	Δ*F*	*p*	
		0.06	0.71	0.09	
Predictors	Predictor statistics				
	*β*	*b*	SE*_b_*	*t*	*p*
**Baseline Asym**	**0.61**	**0.78**	**0.25**	**3.13**	**< 0.001**
Child Age	−0.15	−0.02	0.03	−0.79	0.30
SUR	0.17	0.01	0.01	0.75	0.67
NEG	−0.02	0.00	0.02	−0.10	< 0.05
RCO	0.18	0.03	0.03	0.92	0.07
	Model statistics: Predicting Mask 2 asymmetry (boys)				
		Δ*R* ^2^	Δ*F*	*p*	
		0.03	0.36	< 0.05	
Predictors	Predictor statistics				
	*β*	*b*	SE*_b_*	*t*	*p*
**Baseline Asym**	**0.68**	**1.02**	**0.28**	**3.66**	**< 0.001**
Child Age	0.04	0.01	0.06	0.18	0.86
SUR	−0.08	−0.02	0.05	−0.45	0.66
NEG	0.19	0.04	0.04	0.97	0.35
RCO	0.05	0.01	0.05	0.21	0.83
	Model statistics: Predicting Mask 2 asymmetry (girls)				
		Δ*R* ^2^	Δ*F*	*p*	
		0.25	3.72	< 0.05	
Predictors	Predictor statistics				
	*β*	*b*	SE*_b_*	*t*	*p*
Baseline Asym	0.22	0.29	0.25	1.16	0.26
**Child Age**	**−0.41**	**−0.06**	**0.03**	**−2.14**	**< 0.05**
SUR	−0.27	−0.02	0.01	−1.25	0.22
NEG	0.46	0.06	0.02	2.49	0.02
**RCO**	**0.53**	**0.08**	**0.03**	**2.94**	**< 0.05**
Model step	Model statistics: Predicting Mask 3 asymmetry (boys)				
		Δ*R* ^2^	Δ*F*	*p*	
		0.03	0.35	< 0.05	
Predictors	Predictor statistics				
	*β*	*b*	SE*_b_*	*t*	*p*
**Baseline Asym**	**0.63**	**1.11**	**0.32**	**3.43**	**< 0.001**
Child Age	0.14	0.05	0.07	0.73	0.48
SUR	−0.06	−0.02	0.05	−0.36	0.72
NEG	0.18	0.04	0.04	0.93	0.37
RCO	0.12	0.04	0.06	0.58	0.57
	Model statistics: Predicting Mask 3 asymmetry (girls)				
		Δ*R* ^2^	Δ*F*	*p*	
		0.15	1.68	0.20	
Predictors	Predictor statistics				
	*β*	*b*	SE*_b_*	*t*	*p*
**Baseline Asym**	0.05	0.06	0.28	0.22	0.83
Child Age	0.15	0.02	0.04	0.58	0.57
SUR	−0.37	−0.02	0.02	−1.26	0.22
NEG	0.36	0.04	0.03	1.62	0.12
RCO	0.15	0.02	0.03	0.69	0.50
	Model statistics: Predicting Mask 4 asymmetry (boys)				
		Δ*R* ^2^	Δ*F*	*p*	
		0.14	1.30	0.07	
Predictors	Predictor statistics				
	*β*	*b*	SE*_b_*	*t*	*p*
**Baseline Asym**	**0.68**	**1.01**	**0.31**	**3.26**	**< 0.05**
Child Age	0.03	0.01	0.07	0.14	0.89
SUR	−0.32	−0.09	0.06	−1.56	0.14
NEG	−0.30	−0.06	0.04	−1.38	0.19
RCO	0.03	0.01	0.06	0.10	0.92
	Model statistics: Predicting Mask 4 asymmetry (girls)				
		Δ*R* ^2^	Δ*F*	*p*	
		0.39	5.21	< 0.05	
Predictors	Predictor statistics				
	*β*	*b*	SE*_b_*	*t*	*p*
Baseline Asym	−0.06	−0.10	0.29	−0.34	0.74
Child Age	0.27	0.05	0.04	1.29	0.21
**SUR**	**−0.82**	**−0.06**	**0.02**	**−3.88**	**< 0.001**
NEG	0.09	0.01	0.02	0.51	0.61
RCO	0.34	0.06	0.03	1.90	0.07

Abbreviations: SUR—Surgency, NEG—Negative Emotionality, RCO—Regulation/Orienting.

**TABLE 12:** Hierarchical regressions examining temperament subscale predictors of asymmetry by infant sex (H3).

Model step	Model statistics: Predicting Mask 2 asymmetry (girls)				
		Δ*R* ^2^	Δ*F*	*p*	
		0.25	2.67	< 0.05	
Predictors	Predictor statistics				
	*β*	*b*	SE*_b_*	*t*	*p*
**Baseline Asym**	**0.38**	**0.48**	**0.22**	**2.21**	**< 0.05**
Child Age	−0.12	−0.02	0.02	−0.75	0.46
Duration of Orienting	0.30	0.10	0.06	1.67	0.11
Low Intensity Pleasure	−0.25	−0.08	0.06	−1.28	0.21
Soothability	−0.02	−0.01	0.06	−0.11	0.92
**Cuddliness**	**0.57**	**0.20**	**0.07**	**2.99**	**< 0.01**
Model step	Model statistics: Predicting Mask 4 asymmetry (girls)				
		Δ*R* ^2^	Δ*F*	*p*	
		0.21	1.52	< 0.05	
Predictors	Predictor statistics				
	*β*	*b*	SE*_b_*	*t*	*p*
Baseline Asym	−0.13	−0.23	0.33	−0.69	0.50
Child Age	0.31	0.06	0.05	1.28	0.21
Activity	−0.35	−0.14	0.09	−1.62	0.12
Smiling/Laughter	−0.11	−0.03	0.08	−0.39	0.70
High Intensity Pleasure	−0.51	−0.23	0.13	−1.78	0.09
Perceptual Sensitivity	0.12	0.03	0.08	0.41	0.69
Approach	−0.31	−0.12	0.11	−1.10	0.28
Vocal Reactivity	0.08	0.03	0.10	0.25	0.80

## 4 Discussion

Contrary to the first hypothesis (H1) and extant literature (Planalp et al. 2017), there were no significant differences in infant asymmetry observed across any of the four mask presentations, indicating minimal variability in the neurophysiology underlying fear reactivity as the mask presentations unfolded, regardless of the electrode locations along the frontal scalp sites. Given the varying results in frontal asymmetry across different sites during the masks paradigm noted in prior literature (see Diaz and Bell 2012), it is interesting that there were also no significant differences across the three commonly used sites (i.e., F3/F4, Fp1/Fp2, F7/F8) in the present study, indicating that there are likely limited differences in observed asymmetry based on scalp location in the frontal lobe in this sample.

Given the lack of significant differences in frontal asymmetry across this task, it was surprising to see significant differences in the observed regulation and distress as measured via behavioral observations. Our results indicated that infants exhibited more intense distress during the final phase of the mask paradigm and also engaged in more frequent regulatory behaviors. This may be due to the unique nature of the final mask (a gas mask), in that it does not directly portray a human or human‐like face as the other masks do, and thus may be uniquely novel to infants. However, it is also likely that by the end of the task, infants are beginning to lose interest, become fatigued or disturbed by the continuing lack of caregiver engagement. This may in part explain the results of Planalp et al. (2017), wherein they described the mask paradigm as eliciting a progressively increasing fear response. It may be that the mask paradigm does not become increasingly fear‐inducing, but that infants instead become more distressed/need to self‐soothe more toward the end of the episode due to other task‐related factors (i.e., lack of caregiver interaction). It is notable that the observed behavioral changes (i.e., greater distress/more regulation efforts) did not translate into differences in the frontal asymmetry parameters. It may be that the behaviors coded for this study do not implicate the withdrawal/approach network captured by the EEG recording. It may also be that because the task is relatively brief, the behaviors are not occurring long enough to produce pronounced shifts in related neurophysiology.

While baseline asymmetry was predictive of asymmetry observed during the first mask presentation, it was not a substantive predictor of the asymmetry markers ascertained during subsequent mask presentations. This pattern of results may be indicative of an initial trait‐like reaction to the novel stimuli, which was not maintained across the task, providing support for the capability model of frontal alpha asymmetry in infancy. Additionally, asymmetry at the first mask predicted the following two presentations, with asymmetry at the third mask presentation only predictive of the fourth mask's asymmetry. These results indicate that there is predictive utility in the initial response and point to the importance of temporal proximity. The lack of a consistent predictive pattern suggests that infants become accustomed to the mask presentations over the course of the task, leading to gradual neurophysiological shifts that were not evident when examined via ANOVA. It may also indicate that some of these masks are less novel or less emotionally stimulating than others, such as the second mask, which takes the form of an old man and may be more familiar to children who spend time with older adults (i.e., grandparents).

As hypothesized (H2), Surgency was predictive of left frontal activation; however, only for the first mask in the sequence, and in contrast with our hypothesis, it emerged as a predictor of right frontal asymmetry during the final mask presentation. Contrary to our hypotheses, Negative Emotionality was not a significant predictor of right frontal asymmetry but rather emerged as a significant predictor of left frontal asymmetry during the second mask presentation. This pattern of results indicates that while infants higher in Surgency may be more approach‐driven when initially presented with a novel stimulus, they appear to become more withdrawn as the task continues, potentially due to the lack of direct interaction that an infant higher in Surgency may be seeking. Additionally, results obtained for the second mask indicated that infants who were higher in Negative Emotionality, and Fear in particular, exhibited relative left frontal activation. It may be that infants with higher trait scores in these areas were attempting to get their mothers’ attention for comfort and were thus more approach‐motivated in the moment, with the ultimate goal of avoiding the novel stimuli.

As hypothesized (H3), there were notable differences in links between temperament and neurophysiological reactions to fear‐eliciting stimuli as a function of infant age and sex. Negative emotionality emerged as a significant predictor during different mask trials and in different directions with respect to frontal alpha asymmetry, dependent on infant age. Specifically, infants 10 months and older with higher Negative Emotionality, particularly Fear and Sadness, were more likely to show right frontal asymmetry during the final mask presentation, as would be expected for a distressing task. For infants under 10 months of age, Negative Emotionality emerged as a predictor of left frontal asymmetry during the third mask presentation, which may be similarly explained by these infants potentially seeking reassurance from their caregiver. There may be something unique about the 10‐month timepoint with respect to fear development, as prior research has shown particularly rapid growth in reactivity to novelty during this stage in development (Gartstein et al. 2018; Rothbart 1989).

Results of the present study have implications for age points recommended for the Lab‐TAB protocol. That is, “Pre‐Locomotor” is indicated for infants aged 6 months (Goldsmith and Rothbart 1996b), with the “Locomotor” version, which includes a different task to measure fear reactivity (i.e., remote‐controlled spider), recommended for infants aged 12 months (Goldsmith and Rothbart 1996a). Presumably, this change in paradigm to measure reactivity to novelty is introduced because a different set of reactions is anticipated, yet older infants in our sample were already demonstrating a different pattern of reactivity, starting at 10 months of age—more likely to have right frontal asymmetry during the final mask presentation predicted by fear and sadness, whereas younger infants’ Negative Emotionality predicted left frontal asymmetry during the Mask 3 presentation. Also, in the older infant group, Surgency was predictive of left frontal asymmetry during Mask 1; however, for the younger infant group, Surgency was predictive of right frontal asymmetry during the final mask presentation, again contradicting our expectations. It may be that the younger infants are more likely to be distressed during a longer period of inactivity, wherein they are unable to interact with their environment and especially caregivers, as opposed to the older infants who may be more used to waiting brief periods between opportunities to interact/engage with their mothers. These results indicate that the neurophysiology underlying the approach/avoidance elicited by different masks varies by infant age in terms of its associations with temperamental predispositions.

The results that Surgency was predictive of right frontal asymmetry during the final mask presentation for girls are interesting, as boys are commonly rated higher in related attributes (e.g., High Activity Pleasure, Activity; Olino et al. 2013). Given the above interpretation of the role Surgency plays in predicting right frontal asymmetry during the final mask presentation, it may be that girls were more prone to distress when they lacked access to caregiver support over time (i.e., when they were required to sit in a highchair for the duration of the task). On the other hand, the predictive role of Regulation/Orienting for girls is concordant with prior literature, as girls are often described as having better regulatory abilities in childhood (Else‐Quest et al. 2006). With regard to the prediction of left frontal asymmetry during Mask 2, the contribution of Cuddliness may indicate that the infants were attempting to draw their caregiver's attention, so they were in “approach mode,” as infants higher in Cuddliness are those who find being held by their caregiver more enjoyable. It is not entirely clear why this effect emerged for the second mask only and should be replicated. However, it may be significant that this mask, the old man, is the most human‐like and perhaps most familiar to the infants. No significant temperament predictors emerged for the boys in our sample, which may be attributable to the slightly smaller number of boys or due to lower variability in reported temperament traits; boys had smaller ranges for reported traits (e.g., boys Surgency: 24.12–34.95, girls Surgency: 22.74–38.68). It is also plausible that temperament is more critical in shaping neurophysiological response to the mask stimuli for girls—a finding that also requires additional research.

## 5 Limitations, Future Directions, and Conclusions

While this study provides new insights into a frequently utilized laboratory task, it is important to note that there are a number of limitations. Our sample is comparable to prior infant EEG work; however, it is still relatively small, particularly when considering the age and sex analyses described above. Similarly, age and gender analyses are somewhat limited due to the small number of participants in each category, which prohibited us from examining age × sex effects. Age analyses were also limited by the cross‐sectional design. The soothability subscale of the IBQ‐R showed relatively low internal consistency, which may impact a portion of the analyses. Future work would benefit from increased sample sizes and longitudinal designs to enable analyses of developmental and not just age‐based differences in reactivity and regulation. Future work could also focus on more “at‐risk” samples of infants, as infants exposed to greater contextual risk, for example, may respond with different patterns of reactivity and regulation. It may also be important for future work to consider other fear reactivity paradigms from the Lab‐TAB, such as the “stranger approach” or “jumping/remote spider,” to see if there are significant differences in reactivity/regulation during these tasks or portions of these tasks. Finally, it may be interesting to replicate these findings with older children, as they begin to have experiences and a more sophisticated understanding of the cultural context, likely to change perception of the mask paradigm. That is, infants are unlikely to have a sense that these masks are meant to be frightening; however, older children should have more experiences capable of influencing fear reactivity, as well as more advanced cognitive skills to rapidly process relevant information. An older sample would also likely present with more effective regulatory behaviors, becoming less reliant on their caregivers in coping with a fear‐inducing situation.

Of particular interest in this study is the discrepancy between the results of trial comparisons for the EEG asymmetry markers and observed distress/regulation. While a conclusive interpretation of these findings awaits replication and extension, it may be that infants’ behavioral expression occurs first, later translating into brain activity changes. It may also be that changes in behavioral reactions identified in this study are not related to the neurophysiology of approach/avoidance per se and occur largely because of fatigue or inability to directly interact with either the stimuli or their mothers. Thus, by the end of the task, infants present with greater distress/regulation efforts but without activation of the underlying approach or avoidance networks. It may also be that uniform shifts in alpha frontal asymmetry were not observed because responses to each mask and across the episode were largely a function of temperament factors discussed above. If these individual differences play a significant role in shaping infants’ responses, consistent asymmetry shifts across this task would not be identified in the overall sample. Importantly, the present study reiterates the need to further examine not only behavioral responses but also concurrent neurophysiological activation so that we may be able to better understand the mechanisms driving infant reactivity and regulation. This work also underscores the need to study commonly utilized measures of infant temperament more systematically at a fine‐grained level, as unique and potentially important aspects may be missed during broad examinations, averaging across multiple indicators.

## Conflicts of Interest

The authors declare no conflicts of interest.

## Data Availability

The data that support the findings of this study are available from the corresponding author upon reasonable request.
